# Hypertensive Disorders of Pregnancy, Preterm Delivery, and Infant Size: Which Mothers Have Highest Cardiovascular Disease Mortality?

**DOI:** 10.1111/ppe.70033

**Published:** 2025-06-05

**Authors:** Sage Wyatt, Rolv Skjærven, Lars Vatten, Allen J. Wilcox, Aditi Singh, Kari Klungsøyr, Suzan L. Carmichael, Nils‐Halvdan Morken, Rolv Terje Lie, Liv Grimstvedt Kvalvik

**Affiliations:** ^1^ Department of Global Health and Primary Care University of Bergen Bergen Norway; ^2^ Center for Fertility and Health Norwegian Institute of Public Health Oslo Norway; ^3^ Department of Public Health Norwegian University of Science and Technology Trondheim Norway; ^4^ Epidemiology Branch National Institute of Environmental Health Sciences Durham North Carolina USA; ^5^ Department of Health Promotion The Norwegian Institute of Public Health Bergen Norway; ^6^ Department of Pediatrics Stanford University School of Medicine Stanford California USA; ^7^ Department of Obstetrics and Gynecology Stanford University School of Medicine Stanford California USA; ^8^ Department of Obstetrics and Gynecology Haukeland University Hospital Bergen Norway

**Keywords:** CVD, HDP, maternal health, preeclampsia, pregnancy complications

## Abstract

**Background:**

Research on new‐onset hypertensive disorders of pregnancy (HDP) and long‐term maternal cardiovascular disease (CVD) death has focused on mothers of small‐for‐gestational‐age infants rather than large‐for‐gestational‐age infants.

**Objectives:**

We further explored this focus by investigating CVD death in mothers with HDP by gestational age at delivery across the full spectrum of infant birth size.

**Methods:**

We used data from the Medical Birth Registry of Norway, the Norwegian National Population Register, and the Norwegian Cause of Death Registry, with information on mothers giving birth 1967–2020. This data was used to predict CVD death in the decades following pregnancy.

**Results:**

We found the lowest CVD mortality among mothers with no HDP, term delivery, and a first infant with birthweight above average. These women constituted our reference group in the analyses. We found the highest risk of CVD death among mothers with preterm HDP and infants with above average birthweight for gestational age (HR 6.87, 95% CI 4.98, 9.48), not with infants below average birthweight for gestational age (HR 3.06, 95% CI 2.37, 3.93).

**Conclusions:**

There is an interactive association between HDP and large infant birthweight in preterm first births. The high risk associated with the particular combination of HDP, preterm birth, and high infant birthweight for gestational age warrants further research to understand its causal underpinnings.

## 1 Background

A number of pregnancy complications have been associated with the mother's subsequent risk of cardiovascular disease (CVD). For example, new‐onset hypertensive disorders of pregnancy (HDP), including gestational hypertension, preeclampsia, eclampsia and HELLP [1], are associated with an estimated 2–4 fold increased risk in CVD mortality [2]. Other factors associated with long‐term maternal CVD include preterm delivery, small infant size (usually measured as birthweight by gestational age), and lifetime number of births [3, 4, 5, 6, 7]. These associations may persist even when controlling for pre‐gestational cardiovascular risk factors [8].

Previous literature has shown that when HDP, preterm delivery, and small for gestational age (SGA) all occur in the same pregnancy, there is an additive association of increased risk [9, 10, 11, 12, 13]. However, a recent study found that the increase in risk of CVD after an HDP pregnancy was not highest among mothers with an SGA infant, but among mothers with a large‐for‐gestational‐age (LGA) infant [4]. Mothers with LGA infants often have underlying cardiometabolic risk factors such as gestational diabetes or obesity, making a higher risk of CVD plausible [14]. We pursue this association between HDP with large infants and CVD, taking into account the possible role of gestational age, which has not been explored previously. Our purpose is to identify mothers at highest risk of CVD given strata of these three characteristics: HDP, gestational age at delivery, and infant size for gestational age.

## 2 Methods

### 2.1 Study Design

The Medical Birth Registry of Norway (MBRN) is a population‐based compulsory registry of 3 million births to date since 1967, including stillbirths, live births, and spontaneous abortions from 12 to 16 weeks in Norway. In this study, the term ‘mother’ is used to refer to a person who has given birth and includes individuals of all gender identities. The MBRN does not collect data on gender identity, and any individual giving birth is classified as a ‘mother’ regardless of their social role. Information is collected on both infant and maternal health during pregnancy as well as information on delivery and infant outcomes, including vital status, birth weight, gestational age and neonatal diagnoses. Reporting methods underwent significant changes in 1999, such as including gestational age estimation based on ultrasound measurements in addition to last menstrual period and the inclusion of checkboxes for many conditions, including hypertensive disorders of pregnancy. Prior to this date, pregnancy complications were written in the form of free text [15]. Preterm birth registrations had over 99% specificity and sensitivity, and high and low birthweight registrations were verified in 100% of validated cases [16].

**FIGURE 1:**
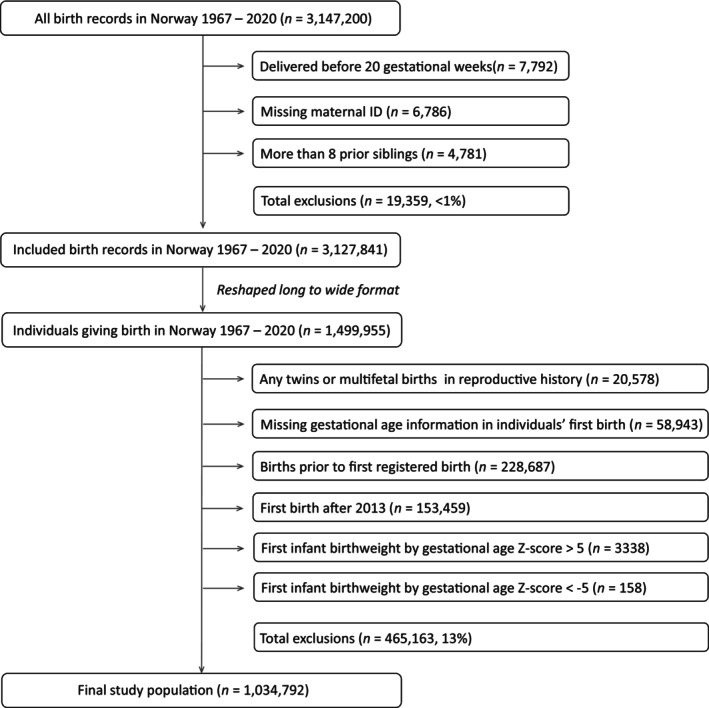
Flow chart of inclusion into study population.

The population for this study consisted of women who had their first birth registered in the MBRN from 1967 to 2013. The lifetime number of births of each mother was captured by including mothers whose first birth was within the MBRN period (from 1967) and with at least 7 years of follow‐up for subsequent births, up to 2020 [17]. Births delivered before 20 gestational weeks were excluded because the definition of HDP is restricted to cases diagnosed after 20 weeks of gestation. Mothers with missing information on gestational age at delivery for their first birth and mothers with more than eight lifetime births were excluded from the analyses. Mothers whose infant was registered with birthweight by gestational age *Z*‐scores greater than 5 or less than −5 were also excluded due to the likelihood of error in registered gestational age or birthweight. Mothers with twin or other multi‐infant births were also excluded, as HDP has a different relationship to gestational age and CVD in these births [18]. A flow chart of included data is shown in Figure 1.

### 2.2 Variable Definitions

The primary exposure in this study was HDP. HDP included gestational hypertension, preeclampsia, eclampsia and HELLP syndrome. Gestational hypertension was defined as hypertension after 20 weeks gestation (at least 140/90 mmHg). Preeclampsia was defined as gestational hypertension combined with proteinuria (≥ 0.3 g or ≥ 1+ reading on a dipstick). Eclampsia was defined as perinatal general convulsions not due to another cause. Haemolysis, elevated liver enzymes and low platelets are acronymized as HELLP [19]. Chronic hypertension with superimposed preeclampsia, eclampsia, or HELLP was included as HDP, but otherwise not considered.

We further stratified HDP diagnosis in the first pregnancy by preterm birth, determined by ultrasound, or by last menstrual period if ultrasound estimates were not available. Preterm delivery was defined as delivery before 37 completed gestational weeks; later deliveries were defined as term. We used birthweight‐by‐gestational age and sex to define infant size. Birthweight *Z*‐scores and percentiles were generated using methods outlined in Skjærven et al. 2000, and were not parity‐specific [20].

We retrieved mortality data by linking the MBRN to the Norwegian Cause of Death Registry using the unique national identity number given to every Norwegian resident. Our main outcome was premature maternal death from cardiovascular disease (CVD), defined as death from ischemic heart disease (ICD 10 codes I20–I25, ICD 7–9 codes 410–414), cerebrovascular disease (ICD 10 codes I60–I69, ICD 7–9 codes 430–438), or peripheral arterial disease (ICD 10 codes I70–I72, I74; ICD7‐9 codes 440–444) in mothers through 69 years of age [21].

### 2.3 Statistical Analysis

We divided infant birth size in the first pregnancy into quartiles of birthweight by gestational age in the total population (< 25th percentile, 25–49th percentile, 50–75th percentile, > 75th percentile).

We also stratified mothers by term and preterm delivery and statistically tested for interaction between birthweight by gestational age and HDP using relative excess risk due to interaction (RERI) [22]. We further display this interaction between two categories of infant birthweight by gestational age and HDP (stratified by gestational age at delivery) on CVD death using generalised linear models.

Our reference group for the main analyses was mothers who had no HDP in their first birth and delivered at term. Cox proportional hazard models were used to assess mothers' mortality through the age of 69 years, defined as premature CVD mortality [23]. Person‐time was measured as years since the mother's last birth until death, emigration, or age 69. CVD mortality was treated as a cause‐specific outcome. Follow‐up time began at the age of first recorded birth (up to 2013) and was censored at 69 years, at emigration, death due to other causes, CVD death, or July 2020, whichever occurred first. Modelled estimates were adjusted for year of mother's first birth (continuous), mothers' age at first birth (categorised as < 20, 20–24, 25–29, 30–34, and > 34 years), and mothers' highest achieved level of education (categorised as less than high school, high school, college/university, and missing). Information on education was collected by linkage to the National Education Database at Statistics Norway.

Additional sensitivity analyses were performed using data on lifetime number of births (1 and 2 or more), and medical intervention for delivery (spontaneous and induced/caesarean). We performed separate analyses for preeclampsia and gestational hypertension to investigate differences in results and robust use of the combined HDP variable. We conducted summary‐the level quantitative bias analyses to test the impact of unmeasured confounding from GDM and high pregestational BMI using the ‘confounder.array’ method from the R package ‘episensr’.

We calculated the prevalence of high BMI (BMI > 30) using data after 2015, because data contained only 10% missing after 2015. In the data from 1967, 92% was missing because data collection on BMI was not introduced until 2006, when electronic birth notification was introduced and had a very gradual increase in reporting prevalence. We calculated the prevalence of GDM in first births after 2016, due to a change in national guidelines in 2017 that maximised accurate diagnosis and consistent prevalence from 2017 to 2020 [24]. Associations between confounders and outcomes were extracted from previous studies, with the association between GDM and CVD mortality estimated to be RR 1.68 [2] and the sex‐adjusted association between high BMI and CVD mortality estimated to be RR 1.81 [25]. Relative risk was used due to the large material and common frequency of the outcome.

### 2.4 Missing Data

Mothers with missing data for gestational age at delivery in the first pregnancy were not included in the study, as shown in Figure 1. Mothers with missing data on education were assigned a separate level in the analysis, including less than high school, high school, college/university and missing data.

### 2.5 Ethics Approval

This research project obtained approval from the Regional Committee for Medical and Health Research Ethics (REK VEST 2015/1728 and REK VEST 13818). Ethical approval did not require informed consent of the participants as no direct participant contact was in the study and all data were de‐identified.

## 3 Results

### 3.1 The Study Sample

**FIGURE 2:**
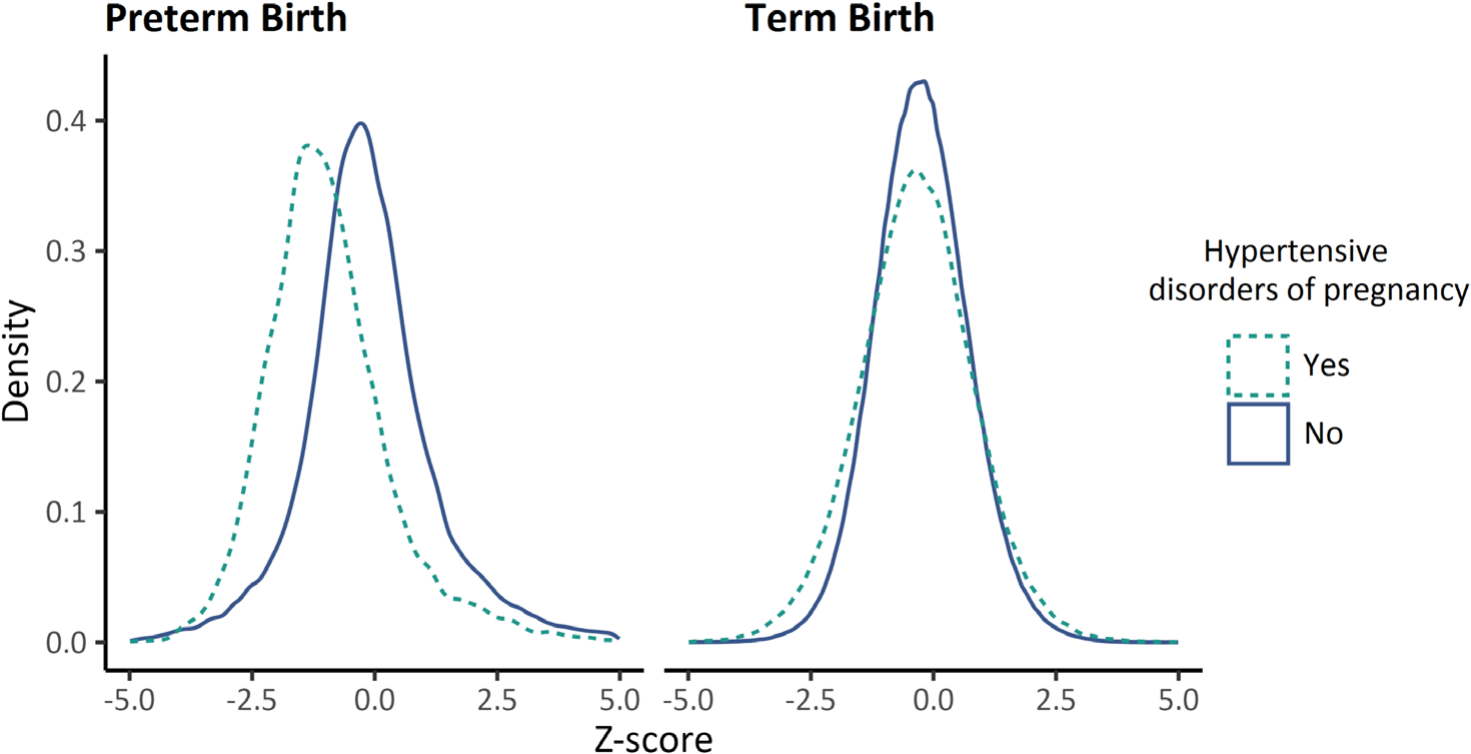
Density plot of birthweight by gestational age *Z*‐score distribution for the first birth, stratified by HDP and gestational age of delivery.

Table 1 shows the characteristics of mothers by HDP status and gestational age at delivery in their first birth. The study included 1,034,792 mothers, of whom 88.7% had a term normotensive first birth. More mothers had term HDP (5.2%) than preterm HDP (1.0). Figure 2 displays the birthweight‐by‐gestational‐age distributions for first‐born infants. For term first‐born infants, the distribution of birthweight‐by‐gestational‐age *Z*‐scores in both strata of HDP was close to gaussian and overlapping, while for preterm deliveries, HDP showed markedly different associations with birthweight by gestational age *Z*‐scores.

**TABLE 1:** Characteristics of mothers given HDP status and gestational age at delivery in the first birth.

	No HDP		HDP	
	Term	Preterm	Term	Preterm
Total number of mothers (% in row)	917,417 (88.7)	53,431 (5.2)	54,095 (5.2)	9849 (1.0)
(% in column)				
Lifetime number of births				
1	151,675 (16.5)	10,853 (20.3)	10,115 (18.7)	2489 (25.3)
2+	765,742 (83.5)	42,578 (79.7)	43,980 (81.3)	7360 (74.7)
Mothers' age at first birth				
< 20	106,072 (11.6)	8352 (15.6)	5233 (9.7)	778 (7.9)
20–24	344,754 (37.6)	18,738 (35.1)	18,812 (34.8)	2906 (29.5)
25–29	300,525 (32.8)	15,758 (29.5)	18,236 (33.7)	3408 (34.6)
30–34	126,613 (13.8)	7520 (14.1)	8467 (15.7)	1833 (18.6)
≥ 35	39,453 (4.3)	3063 (5.7)	3347 (6.2)	924 (9.4)
Mothers' highest achieved education				
< High school	172,049 (18.8)	12,392 (23.2)	9725 (18.0)	1802 (18.3)
High school	351,157 (38.3)	21,242 (39.8)	21,043 (38.9)	3569 (36.2)
University or college	385,184 (42.0)	19,223 (36.0)	22,997 (42.5)	4381 (44.5)
Missing	9027 (1.0)	574 (1.1)	330 (0.6)	97 (1.0)
Year of mothers' first birth				
1967–1979	270,450 (29.5)	16,554 (31.0)	13,307 (24.6)	1412 (14.3)
1980–1989	181,234 (19.8)	10,143 (19.0)	10,972 (20.3)	1668 (16.9)
1990–1999	190,452 (20.8)	11,604 (21.7)	10,838 (20.0)	2454 (24.9)
2000–2011	275,281 (30.0)	15,130 (28.3)	18,978 (35.1)	4315 (43.8)

Thus, for term deliveries, HDP infants' birthweight was centred at the population average (*Z* = 0), while preterm HDP infants' birthweight was centred below average (*Z* = −0.96).

### 3.2 CVD Mortality Varies by HDP Status, Gestational Age at Delivery, and Infant Size

**FIGURE 3:**
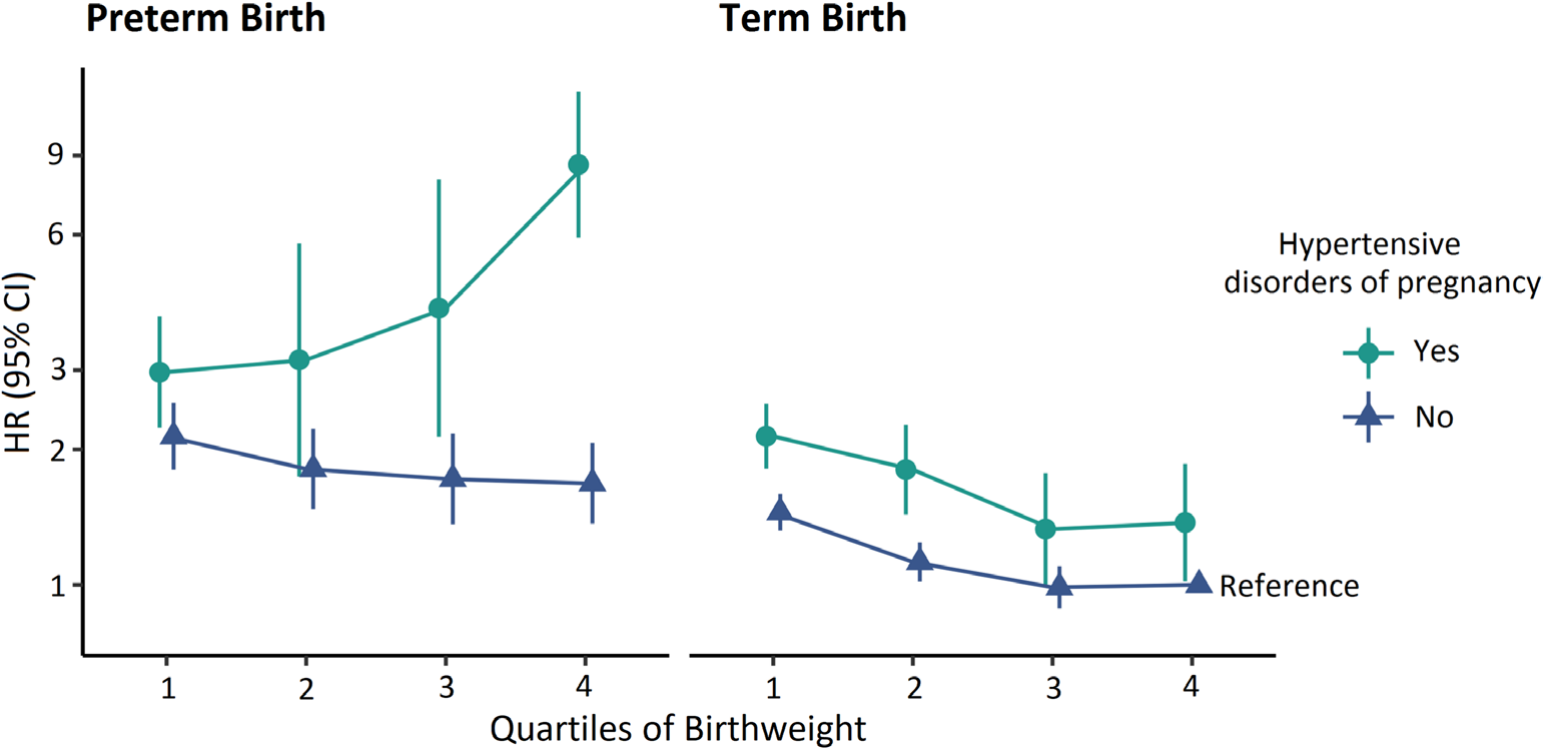
Risk of CVD death across birthweight‐by‐gestational‐age quartiles, gestational age at delivery, and HDP in mothers' first birth. The reference group was mothers whose first birth occurred at term, without HDP, and with an infant birthweight in the highest quartile. Adjusted for year of mother's first birth, mother's age, and mother's education.

Table 2 shows maternal risk of CVD death by quartile of birthweight‐by‐gestational age Z‐score among mothers with and without HDP in their first birth, stratified by term and preterm delivery, additionally visualised in Figure 3. The reference group was mothers whose first birth occurred at term, without HDP, and with an infant birthweight in the highest quartile. Mothers with term delivery or normotensive preterm in their first pregnancy had decreasing risk of CVD death with increasing infant size. In contrast, mothers with preterm HDP had a sharp increase in risk of CVD death with larger infant size evident after the second quartile. This trend was specific to preterm HDP and not normotensive preterm delivery, with highest risk in mothers with preterm HDP and infants above average birthweight by gestational age (HR 6.87, 95% CI 4.98, 9.48). Mothers of infants born with average or below birthweight by gestational age and with preterm HDP had more moderately increased risk, in comparison (HR 3.06, 95% CI 2.37, 3.93). There was evidence of strong multiplicative interaction between HDP and birthweight by gestational age quartiles on CVD mortality for preterm births (RERI = 1.99, 95% CI = 0.92, 3.06), unlike for term births (RERI = −0.27, 95% CI = −0.55, 0.00).

**TABLE 2:** Risk of CVD death across birthweight‐by‐gestational‐age, gestational age at delivery, and HDP in mothers' first birth.

	Mean and below infant birthweight by gestational age		Above mean infant birthweight by gestational age	
	CVD deaths/person‐years	HR^a^ (95% CI)	CVD deaths/person‐years	HR^a^ (95% CI)
No HDP				
Term	3392/13,747,513	1.32 (1.24, 1.41)	1369/775,190	1.00 (Reference)
Preterm	287/8,385,153	2.03 (1.78, 2.30)	194/745,311	1.71 (1.48, 2.09)
HDP				
Term	274/569,343	2.03 (1.78, 2.31)	97/458,624	1.33 (1.07, 1.64)
Preterm	63/159,100	3.06 (2.37, 3.93)	38/37,175	6.87 (4.98, 9.48)

^a^ Adjusted for year of mother's first birth, mother's age, and mother's education.

### 3.3 Sensitivity Analyses

Table 3 shows CVD death risk estimates given HDP, preterm or term delivery, and infant size, stratified by lifetime number of births. Mothers with two or more births, normotensive term first pregnancies and infants' birthweights above average were the reference group. The average birthweight by gestational age was defined as 0 in the population‐level sex‐stratified *Z*‐score. Except for one group, all mothers had the highest CVD mortality with average or below infant birthweight by gestational age. The exception was mothers with preterm HDP, who instead had the highest risk with an above average size infant, with high risk in both mothers with two or more lifetime births and one lifetime birth.

**TABLE 3:** Risk of CVD death stratified by lifetime number of births across birthweight‐by‐gestational‐age, gestational age at delivery, and HDP in mothers' first birth.

	Mean and below infant birthweight by gestational age		Above mean infant birthweight by gestational age	
	CVD deaths/person‐years	HR^a^ (95% CI)	CVD deaths/person‐years	HR^a^ (95% CI)
2+ lifetime births				
No HDP				
Term	2555/11,164,576	1.32 (1.23, 1.42)	1050/6,923,495	1.00 (Reference)
Preterm	200/577,102	1.99 (1.71, 2.31)	132/445,759	1.63 (1.35, 1.95)
HDP				
Term	183/613,233	1.91 (1.63, 2.24)	60/362,120	1.17 (0.90, 1.52)
Preterm	37/113,985	2.81 (2.03, 3.90)	19/26,662	5.54 (3.53, 8.74)
1 lifetime birth				
No HDP				
Term	837/2,582,937	1.54 (1.41, 1.69)	319/1,461,658	1.17 (1.04, 1.34)
Preterm	87/168,209	2.46 (1.96, 3.07)	62/123,584	2.23 (1.72, 2.89)
HDP				
Term	91/161,957	2.68 (2.15, 3.32)	37/96,504	1.99 (1.43, 2.77)
Preterm	26/45,115	3.98 (2.69, 5.88)	19/10,513	10.12 (6.42, 15.93)

^a^ Adjusted for year of mother's first birth, mother's age, and mother's education.

We found no major differences in the results when analysing gestational hypertension and preeclampsia separately (Table S1). We found that mothers with induced or caesarean section preterm births had higher CVD mortality than spontaneous preterm births. One quarter of these mothers had only one lifetime birth (Table S2). Bias from unmeasured confounding was estimated to be < 10% in all levels of exposure. While bias was highest in the group of mothers with highest risk when accounting for unmeasured gestational diabetes mellitus, the group with highest risk of CVD death was the third most biased estimate when considering high pregestational body mass index. No estimates had notably large changes in magnitude or direction (Table S3).

## 4 Comment

### 4.1 Principal Findings

Large infant size emerges as a strong risk factor of CVD death among mothers with preterm HDP. This risk is not confined to mothers with extremely large infants, but rather appears to increase gradually, starting from below the mean. Previous research on birthweight and maternal CVD risk has focused on infants born small for gestational age [9, 10, 11]. Having a large for gestational age offspring in itself has not typically been considered a risk factor for long‐term maternal CVD, given the modest risk of CVD among mothers who deliver large infants at term [26]. HDP, preterm birth, and infant size beyond small for gestational age have been studied in many combinations [3, 4, 27] but we know of no study that considers all three factors in prediction of long‐term CVD death.

### 4.2 Strengths of the Study

This study uses a longitudinal dataset to study perinatal issues in a large population, providing the opportunity to link successive pregnancies. The risk of selection bias in the study design is limited, due to compulsory registration. The large number of observations and long time period are ideal for studying rare outcomes across the life course.

### 4.3 Limitations of the Data

One weakness of our study is that we are limited by the variables recorded in the birth registry. The MBRN is lacking in richness of clinical and behavioural variables. For births during the first 30–40 years of our study, the registry lacks information on biometric and lifestyle information relevant for long‐term outcomes. This makes it challenging to investigate potential underlying biological mechanisms for our findings. Another limitation is although gestational hypertension and preeclampsia registrations have high positive predictive values (preeclampsia diagnosis was confirmed in 88% of births and gestational hypertension was confirmed in 68% of births), their sensitivity is rather poor [28, 29]. Underreporting of mild preeclampsia in pregnancy was, for example, common before 1999, when pregnancy complications were reported as free text [15]. Also, it would be desirable to have information about the timing of onset of pregnancy complications, rather than only the status recorded at birth. However, detailed data on onset of HDP is not available. Some of the subgroups in sensitivity analyses had very few observations and should be interpreted with caution. The number of missing data was low, with 3.8% missing data on preterm birth, 4.1% missing data on birthweight, and 1.0% missing data on mother's education, as shown in Figure 1 and Table 1. It is unpredictable how these missing data may create bias in the results. Prevalence information for GDM and BMI in the quantitative bias analysis were derived from data with a narrow time frame and a large proportion of missing data which may not fully capture the effects of confounding.

### 4.4 Interpretation

The differences in risk prediction by characteristics in the first birth of mothers with HDP could represent etiological differences in unidentified subtypes of HDP. For example, small infant size might be driven by HDP‐associated placental arterial development, whereas large infant size might be driven by maternal cardiometabolic factors [27, 30, 31]. Our findings support the hypothesis of etiological differences between early and late onset HDP, as the association of large infant size with maternal CVD risk was found only among mothers with preterm HDP [27, 32, 33]. Possible etiological differences between early and late onset HDP are still debated [34, 35, 36], but the distinct differences in long‐term maternal risk that we observed seem robust and not likely to be due to chance. Our results are based on the timing of delivery rather than the timing of onset of HDP, which may be influenced by many other factors than HDP such as clinical handling or premature rupture of membranes. Much like our other covariates, lifetime number of pregnancies and medical intervention for delivery, preterm delivery is ultimately an indicator of severity of the HDP condition. While this paper is focusing on first pregnancy only, other papers have examined the lifetime number of complicated pregnancies, which are an important factor in predicting CVD risk [7]. Patterns explored in this paper could also be directions for future research across lifetime reproductive history.

Preeclampsia is a heterogeneous condition in which various pathophysiological mechanisms may converge into a single clinical syndrome. Many attempts have been made to identify preeclampsia subtypes, for example, by whether the condition has a cardiometabolic or a placental origin, whether onset occurs early or late in pregnancy, or whether the underlying pathology originates in the mother or infant [37]. Roberts et al. have called for a re‐evaluation of these dichotomizations, suggesting that specific, clinically relevant subtypes of HDP would be more useful [30]. Likewise, there are various theories of underlying maternal health conditions causing HDP, for example, maternal risk factors for cardiovascular disease [38], failure of the maternal immune system [39], or a healthy response to pregnancies with high infant demands [18].

HDP is known to be associated with later development of type 2 diabetes, especially when co‐occurring with preterm birth and small infant size [40, 41]. While diabetes is independently associated with HDP [42], the evidence so far suggests that subclinical metabolic dysfunction in the upper‐normal range is also associated with HDP [4, 43, 44]. Other data sources besides the MBRN may be more relevant to address questions that require more detailed clinical and biochemical measurements. It would be useful for future studies to explore the high‐risk subtype of HDP that we have identified and specific health conditions linked to it, for example, the high prevalence of GDM in the group of mothers at highest risk for CVD death. Future research on this high‐risk group should be conducted as the prevalence of preterm HDP increases over time [15]. While we observed a higher risk of CVD death in mothers with preterm HDP than in normotensive mothers regardless of lifetime number of births, it is also more common for mothers with preterm HDP than for normotensive mothers to have only 1 lifetime birth. The association between lifetime number of births and underlying cardiometabolic health may be an essential topic in future investigations [45].

### 4.5 Conclusions

In fertile Norwegian women, CVD mortality was highest in mothers with preterm HDP and a normal‐to‐large size infant—an association not seen in term HDP pregnancies or in pregnancies without HDP. Studies of CVD mortality related to pregnancy outcomes usually focus on the risk seen in mothers of small‐for‐gestational‐age infants. Our analysis shows that mothers with preterm HDP and large babies may have a clinically important subtype of preeclampsia that deserves further exploration, especially regarding the association with diabetes mellitus.

## Author Contributions

R.S. and A.J.W. drafted study concept and initiated analyses. S.W. conducted the analyses and wrote the manuscript. S.W. created the figures with the help of A.S. A.S. also contributed to the analysis. N.H.M. offered clinical expertise and insights. The other co‐authors made significant contributions to study concept and manuscript revisions. L.G.K. supervised final writing and analysis.

## Conflicts of Interest

The authors declare no conflicts of interest.

## Supporting information


Data S1.

## Data Availability

The data that support the findings of this study are available from The Medical Birth Registry of Norway. Restrictions apply to the availability of these data, which were used under licence for this study. Data are available from https://helsedata.no/en/forvaltere/norwegian‐institute‐of‐public‐health/medical‐birth‐registry‐of‐norway‐mbrn/ with the permission of The Medical Birth Registry of Norway.
