# Supplementary material for: Hypertensive Disorders of Pregnancy, Preterm Delivery, and Infant Size: Which Mothers Have Highest Cardiovascular Disease Mortality?
Source: Paediatr Perinat Epidemiol. 2025 Jun 5;39(6):515–23. doi: 10.1111/ppe.70033 (PMC12391857; doi:10.1111/ppe.70033)
Supplement: Supplementary file 1 — Data S1. [file PPE-39-515-s001.docx]

Supplementary Table 1. Risk of CVD death stratified by preeclampsia and gestational hypertension across birthweight-by-gestational-age, gestational age at delivery, and HDP in a mothers’ first birth.

|  |  | **Mean and Below Infant Birthweight by Gestational Age** | | **Above Mean Infant Birthweight by Gestational Age** | |
| --- | --- | --- | --- | --- | --- |
|  |  | CVD Deaths/  Person-years | HR^a^  (95%CI) | CVD Deaths/  Person-years | HR^a^  (95%CI) |
| Preeclampsia | | | | | |
| No Preeclampsia | Term | 3483/  14004396 | 1.33  (1.25, 1.42) | 1401/  8553909 | HR 1.00 (Reference) |
|  | Preterm | 294/  762837 | 2.04  (1.80, 2.32) | 204/  576796 | 1.78  (1.54, 2.07) |
| Preeclampsia | Term | 185/  518726 | 2.06  (1.77, 2.41) | 64/  287453 | 1.46  (1.14, 1.87) |
|  | Preterm | 55/  139761 | 3.01  (2.30, 3.95) | 28/  29501 | 6.74  (4.64, 9.80) |
| Gestational Hypertension | | | | | |
| No Gestational Hypertension | Term | 3579/  14266737 | 1.33  (1.25, 1.41) | 1432/  8675074 | HR 1.00  (Reference) |
|  | Preterm | 341/  877237 | 2.11  (1.88, 2.38) | 222/  598596 | 1.87  (1.63, 2.16) |
| Gestational Hypertension | Term | 89/  256385 | 1.92  (1.55, 2.37) | 33/  168694 | 1.12  (0.79, 1.58) |
|  | Preterm | 8/  17374 | 3.22  (1.61, 6.45) | 10/  7701 | 7.09  (3.81, 13.22) |

^a^ Adjusted for year of mother’s first birth, mother’s age, and mother’s education.

Supplementary Table 2. Risk of CVD death stratified by medical intervention for delivery across birthweight-by-gestational-age, gestational age at delivery, and HDP in mothers’ first birth.

|  |  | **Infant Birthweight by Gestational Age equal to or less than the mean** | | | **Infant Birthweight by Gestational Age above the mean** | | |
| --- | --- | --- | --- | --- | --- | --- | --- |
|  |  | CVD Deaths/  Person-years | HR^a^  (95%CI) | % within group with one lifetime birth | CVD Deaths/  Person-years | HR^a^  (95%CI) | % within group with one lifetime birth |
| No HDP | Term | 3392/  13748021 | 1.32  (1.24, 1.41) | 17.0 | 1369/  8385008 | 1.00 (Reference) | 15.7 |
|  | Spontaneous Preterm | 229/  610212 | 1.86  (1.62, 2.14) | 19.8 | 157/  497823 | 1.54  (1.30, 1.82) | 18.9 |
|  | Induced or Cesarean Preterm | 58/  135159 | 3.39  (2.42, 4.77) | 24.1 | 37/  71504 | 3.39  (2.41, 4.77) | 23.8 |
| HDP | Term | 274/  775150 | 1.43  (1.29, 1.59) | 18.8 | 97/  458544 | 1.05  (0.91, 1.21) | 18.6 |
|  | Spontaneous Preterm | 34/  61214 | 3.24  (2.48, 4.24) | 23.7 | 16/  19603 | 3.47  (2.49, 4.84) | 22.7 |
|  | Induced or Cesarean Preterm | 29/  97991 | 2.79  (1.93, 4.04) | 26.3 | 22/  17599 | 11.53  (7.56, 17.58) | 25.9 |

^a^ Adjusted for year of mother’s first birth, mother’s age, and mother’s education.

Supplementary Table 3. Risk of CVD death across birthweight-by-gestational-age, gestational age at delivery, and HDP in mothers’ first birth, adjusted for unmeasured confounding factors.

|  |  | **Mean and Below Infant Birthweight by Gestational Age** | | | **Above Mean Infant Birthweight by Gestational Age** | | |
| --- | --- | --- | --- | --- | --- | --- | --- |
| High Body Mass Index (BMI) | | | | | | | |
|  |  | % with high BMI | Adjusted RR^a^ | % Bias | % with high BMI | Adjusted RR^a^ | % Bias |
| No HDP | Term | 18.3 | 1.35 | -2.2 | 21.4 | 1.00 (Reference) | |
|  | Preterm | 23.4 | 2.00 | 1.4 | 26.6 | 1.65 | 3.6 |
| HDP | Term | 27.8 | 1.94 | 4.4 | 32.8 | 1.23 | 7.9 |
|  | Preterm | 32.7 | 2.84 | 7.8 | 32.3 | 6.39 | 7.5 |
| Gestational Diabetes Mellitus (GDM) | | | | | | | |
|  |  | % with GDM | Adjusted RR^b^ | % Bias | % with high GDM | Adjusted RR^b^ | % Bias |
| No HDP | Term | 4.1 | 1.32 | -0.6 | 5.0 | 1.00 (Reference) | |
|  | Preterm | 4.6 | 2.03 | -0.2 | 7.1 | 1.68 | 1.4 |
| HDP | Term | 6.6 | 2.01 | 1.1 | 7.4 | 1.31 | 1.6 |
|  | Preterm | 7.5 | 3.00 | 1.7 | 13.1 | 6.52 | 5.3 |

^a^ Adjusted for unmeasured confounding from high pregestational body mass index (BMI > 30 Magnitude of confounder-outcome association estimated to be 1.81.

^b^ Adjusted for unmeasured confounding from gestational diabetes mellitus. Magnitude of confounder-outcome association estimated to be 1.68.
